# Relationship between cerebrospinal fluid circulation markers, brain degeneration, and cognitive impairment in cerebral amyloid angiopathy

**DOI:** 10.3389/fnagi.2025.1549072

**Published:** 2025-04-22

**Authors:** Li Zhao, Lingyun Liu, Miao Lin, Linyun Xie, Hui Hong, Qingze Zeng, Shuyue Wang, Ruiting Zhang, Zhenhua Zhao, Peiyu Huang, on behalf of the Alzheimer’s Disease Neuroimaging Initiative

**Affiliations:** ^1^Department of Radiology, Shaoxing People’s Hospital, Shaoxing, China; ^2^Department of Radiology, The Second Affiliated Hospital, Zhejiang University School of Medicine, Hangzhou, China; ^3^Department of Radiology, Shaoxing Maternity and Child Health Care Hospital, Shaoxing, Zhejiang, China

**Keywords:** cerebral amyloid angiopathy, diffusion tensor imaging, perivascular space, Alzheimer’s disease, white matter hyperintensities, cognition

## Abstract

**Objectives:**

To investigate whether cerebrospinal fluid (CSF) circulation markers alter in patients with probable cerebral amyloid angiopathy (pCAA) and whether they are associated with brain degeneration and cognitive impairment.

**Methods:**

We screened pCAA patients from the ADNI3 database according to the Boston 2.0 Criteria. Fifty-two patients with cognitive impairment (26 pCAA; 26 age-sex-matched non-pCAA) and 26 age-sex-matched cognitively normal control (NC) were included in this study. All participants underwent neurological MRI and cognitive assessments. Choroid plexus (ChP) was segmented using a deep learning-based method and its volume was extracted. Diffusion tensor imaging analysis along the perivascular space (DTI-ALPS) was used to assess perivenous fluid mobility. AD pathological markers (Aβ and tau) were assessed using positron emission tomography. Brain parenchymal damage markers included white matter hyperintensities (WMH) volume and brain atrophy ratio. All markers were compared among the three groups. Correlations among the ChP volume, DTI-ALPS index, parenchymal damage markers, and cognitive scales were analyzed in the pCAA group.

**Results:**

The three groups exhibited significant differences in cognitive scores, AD biomarkers, and imaging markers. *Post hoc* analyses showed that patients with pCAA had significantly higher WMH volume, higher Aβ and tau deposition, and lower DTI-ALPS compared to NC. However, no difference in ChPs volume was found among the groups. Controlling for age, sex, and vascular risk factors, partial correlation analyses showed a significant negative correlation between the DTI-ALPS and WMH volume fraction (*r* = −0.606, *p* = 0.002). ChP volume was significantly associated with the Montreal cognitive assessment score (*r* = −0.492, *p* = 0.028).

**Conclusion:**

CSF circulation markers were associated with elevated WMH burden and cognitive impairments in probable CAA.

## Introduction

Cerebral amyloid angiopathy (CAA) is a specific type of small vessel disease defined by β-amyloid (Aβ) deposition in cortical and leptomeningeal vessels (Charidimou et al., 2022; Beaman et al., 2022; Koemans et al., 2023; Greenberg and van Veluw, 2024). Aβ deposition may causes various detrimental effects, including vessel wall thickening and stiffening, loss of vascular smooth muscle cells, blood–brain barrier disruption, etc. (Beaudin et al., 2022; Deng et al., 2022; Chen et al., 2023; Fotiadis et al., 2016, 2021; Greenberg et al., 2020). These pathological factors can further lead to hemorrhagic or ischemic events and brain degeneration (Charidimou et al., 2019; Beaman et al., 2022; Wang et al., 2024; Rabin et al., 2022).

In recent years, the development of the brain waste clearance theory has provided new insights into the mechanisms of CAA-related brain degeneration (Nedergaard and Goldman, 2020). The choroid plexus (ChP) is the primary site of cerebrospinal fluid (CSF) secretion (Wichmann et al., 2021). CSF flows through the ventricles and subarachnoid spaces, further entering the perivascular spaces and brain parenchyma, where it flushes out metabolic waste (Jessen et al., 2015). Previous pathological and animal studies have found that Aβ may deposit in the ChP (Sasaki et al., 1997), inducing cellular toxicity and apoptosis (Vargas et al., 2010). Additionally, since vessel pulsatility is considered a major driving factor of CSF flow (Xie et al., 2024), Aβ deposition in cortical and leptomeningeal vessels may disrupt vessel wall movement and reduce CSF mobility. This can lead to stagnation of interstitial fluid (ISF), accumulation of toxic substances in the parenchyma, and subsequent neurodegeneration (Durrani et al., 2023; Chen et al., 2022; Kim et al., 2020; van Veluw et al., 2024b). This theory offers a new perspective for understanding the progression of brain degeneration in CAA (van Veluw et al., 2024a). However, as most previous studies were conducted in rodent models, there is a critical need for clinical translational research.

Magnetic resonance imaging (MRI) provides a clear visualization of ChP structure. Previous studies have found that ChP volume increases with aging and in various neurodegenerative diseases (Čarna et al., 2023; Jeong et al., 2023), potentially indicating pathological hypertrophy and decreased CSF production. However, no *in vivo* investigations have been conducted to examine ChP changes in CAA. Diffusion tensor imaging analysis along the perivascular space (DTI-ALPS) (Taoka et al., 2017) is a method used to evaluate fluid motion in the perivenous spaces. Research has demonstrated a strong correlation between DTI-ALPS and various neurological disorders (Hong et al., 2024; Jiang et al., 2023). Furthermore, DTI-ALPS can predict long-term outcomes in patients with Alzheimer’s disease (AD) (Huang et al., 2024), Parkinson’s disease (Zhou et al., 2024), and other conditions. Utilizing these CSF circulation markers may help elucidate the association between waste clearance and brain degeneration in CAA.

The definite diagnosis of CAA relies on histopathological analysis from brain autopsy or biopsy samples. Nonetheless, probable diagnosis could be inferred according to the Boston criteria, based on clinical and MRI information. In 2022, the Boston criteria were updated to version 2.0 (Charidimou et al., 2022), which demonstrated superior accuracy compared to the older version. To be specific, the Boston criteria 2.0 classify CAA into different diagnostic certainty levels: definite CAA, probable CAA with supporting pathology, probable CAA (pCAA), and possible CAA. The first two categories require full postmortem brain examination or biopsy confirmation. The pCAA is diagnosed based on combined clinical and imaging criteria. Clinical manifestations include spontaneous intracerebral hemorrhage, transient focal neurological episodes, or cognitive impairment/dementia (Perini et al., 2023). Imaging criteria require either: (1) at least two strictly lobar hemorrhagic lesions on T2*-weighted MRI, or (2) one lobar hemorrhagic lesion plus one white matter feature (either severe centrum semiovale perivascular spaces or multifocal white matter hyperintensities). Although the diagnosis of pCAA is not definitive, it provides a valuable framework for clinical research on this important disease.

In this study, we aimed to: (1) determine whether the two CSF circulation markers were altered in patients with pCAA as defined by the new Boston 2.0 criteria, and (2) examine the associations between CSF circulation markers, brain degeneration, and cognitive impairments. We hypothesized that patients with probable CAA exhibit altered CSF circulation markers, which may contribute to brain degeneration.

## Methods and materials

### Participants

The data used in this article were from the Alzheimer’s Disease Neuroimaging Initiative (ADNI) database.^1^ The database was launched by the National Institute on Aging (NIA), the Food and Drug Administration (FDA), and the National Institute of Biomedical Imaging and Bioengineering (NIBIB), aiming to explore whether serial MRI, positron emission tomography (PET), biological markers, and other neuropsychological assessment can be used for early detecting and tracking AD. Participant inclusion and exclusion criteria are available at www.adni-info.org. All procedures performed in studies involving human participants were under the ethical standards of the Institutional and National Research Committee and with the 1964 Helsinki Declaration and its later amendments or comparable ethical standards. Written informed consent was from all participants and authorized representatives before any protocol-specific procedures were carried out in the ADNI study. More details can be found at http://www.adni-info.org.

**Figure 1:**
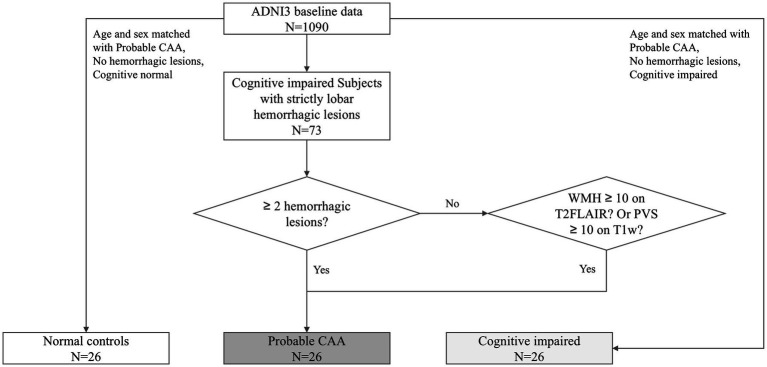
Subject enrollment flowchart. CAA, cerebral amyloid angiopathy.

The diagnosis of pCAA was based on the Boston Criteria version 2.0 (Figure 1). A radiologist with over 10 years of diagnostic experience evaluated the entire ADNI3 baseline dataset, identifying lobar hemorrhagic lesions on T2* images. According to the Boston Criteria, subjects with cognitive impairments and either more than two lobar hemorrhagic lesions or one lobar hemorrhagic lesion plus one white matter feature (e.g., severe perivascular spaces in the centrum semiovale or white matter hyperintensities in a multispot pattern) were classified as pCAA. Since the diagnosis of probable CAA also requires clinical impairment, we included only those with cognitive impairment. To control for the potential influence of cognitive status, which has been associated with CSF circulation abnormalities, we also included a group with MCI but without CAA (MCI-nonCAA). Finally, 26 pCAA cases, 26 age- and sex-matched MCI-nonCAA, along with 26 age- and sex-matched normal controls (NC) were included in this study. The groups were matched using the propensity score matching tool in SPSS Statistics Version 27.0 (IBM).

#### Demographic and clinical data

Demographic and clinical data were obtained from the ADNI database,^2^ including age, sex, smoking status, and vascular risk factors such as hypertension, diabetes, hyperlipidemia, stroke, myocardial infarction, and atrial fibrillation. A summary vascular risk factor score (VRFs) was calculated by summing the presence of each factor.

#### Neuropsychological assessment

The neuropsychological assessment included the mini-mental state examination (MMSE), Montreal cognitive assessment (MoCA), and clinical dementia rating scale sum of boxes (CDR-SOB).

#### PET acquisition and preprocessing

The amyloid PET scans were performed using two tracers (florbetapir, or FBP; florbetaben, or FBB). For this study, we analyzed amyloid PET data processed by UC Berkeley and Lawrence Berkeley National Laboratory. The summary standardized uptake value ratio (SUVR) was calculated by averaging the uptake values across four primary cortical regions (frontal, anterior/posterior cingulate, lateral parietal, and lateral temporal) and normalizing by the whole cerebellum. To promote the harmonization of Aβ PET imaging, we utilized data from FBB and FBP to derive standardized Centiloid (CL) conversions, as previously described (Royse et al., 2021).

The tau PET scans were performed using one tracer (flortaucipir, or FTP). We also analyzed tau PET data processed by UC Berkeley and Lawrence Berkeley National Laboratory. The SUVR for the temporal meta-ROI was determined as a volume-weighted average of the SUVRs from the entorhinal cortex, amygdala, parahippocampal gyrus, fusiform gyrus, inferior temporal cortex, and middle temporal cortex. The detailed acquisition protocols are described in the ADNI PET Technical Procedures Manual.^3^

#### Magnetic resonance imaging acquisition

The imaging data from the ADNI were acquired from multiple centers utilizing standardized protocols. We include 3D T1-weighted (T1w), 3D T2 FLAIR, axial T2*-weighted, and axial diffusion tensor imaging (DTI) sequence. All imaging data were obtained using 3T scanners manufactured by three leading vendors (GE, Siemens, and Philips). Detailed information on the parameters can be found at https://adni.loni.usc.edu/methods/documents/mri-protocols/.

#### Brain segmentation

We performed brain segmentation in each subject using the “recon-all” processing pipeline in FreeSurfer (version 6.0).^4^ It segmented different tissue types and estimated the total intracranial volume (TIV). We calculated the brain parenchymal-to-TIV ratio to reflect brain atrophy.

#### WMH assessment and segmentation

T2 FLAIR images were used for WMH assessment. Visual assessment was performed using the Fazekas grading (Fazekas et al., 1993). For volume quantification, an automatic segmentation tool (Lesion Segmentation Tool) based on the lesion prediction algorithm of the Statistical Parametric Mapping software (SPM12)^5^ was used. The automatically created WMH labeled images were then manually corrected for misclassified tissues. WMH volume was extracted and normalized using the subject’s TIV.

#### Choroid plexus segmentation

For each subject, the 3D FLAIR image was registered to the 3D T1w image using linear registration. The ChP was then segmented based on both 3D T1w and 3D FLAIR images using an in-house-trained deep learning model. This model was trained using 44 subjects scanned with various scanners and tested on 15 subjects. Compared with manual labels, the model demonstrated excellent performance on the testing dataset, achieving a mean Dice coefficient of 0.878, a mean precision of 0.872, and a mean recall of 0.888. Visual inspection was performed to ensure segmentation quality. The ChP volume was then calculated and normalized using the subject’s total intracranial volume (TIV).

#### DTI-ALPS evaluation

The processing of DTI data was conducted using FSL 6.0^6^ and MRtrix3.^7^ Preprocessing steps included Gibbs ringing removal, eddy-current and head motion correction, and bias field correction. Then, the b0 and b1000 images were used to fit an ellipsoid model using the DTIFIT command in FSL. All the generated results including the fractional anisotropy (FA) map and the mean diffusivity (MD) map were normalized to the MNI space using linear registration. Regions of interest (ROI) containing 5 voxels (40mm^3^) were placed at the projection and association fiber regions (Zhang Y. et al., 2021) based on the anisotropy color maps and then the diffusivities at the x, y, and z orientations were extracted. The DTI-ALPS index was calculated using the following formula: DTI-ALPS index, mean (Dxproj, Dxassoc) / mean (Dyproj, Dzassoc). The DTI-ALPS indices in the bilateral hemispheres were calculated, respectively, and the mean of them was used in further analyses. To reduce site effects, we conducted multicenter data harmonization on DTI-ALPS using the COMBAT method (Fortin et al., 2017).

### Statistical analysis

All statistical analyses were performed using SPSS Statistics Version 27.0 (IBM) and R (Version 4.2.1). Mean ± SD and median (interquartile range) were reported for normally and non-normally distributed variables, respectively. Frequencies (%) were presented for categorical variables. A *p*-value <0.05 was considered statistically significant.

We first analyzed whether demographic, clinical, imaging, and disease markers differed among the three groups. Continuous variables were analyzed using ANOVA, categorical variables using Chi-square tests, and ordinal variables using Kruskal-Wallis tests. Post-hoc analyses were conducted to identify sources of differences.

Second, we performed Spearman’s correlation analyses to examine the associations between CSF circulation markers and brain parenchymal damage, AD pathological markers, and cognitive impairments in the pCAA group. Age and sex were included as covariates. Considering the possible influence of vascular risk factors on CSF circulation and brain degeneration, we also performed additional correlation analyses while controlling the VRFs.

## Results

**Figure 2:**
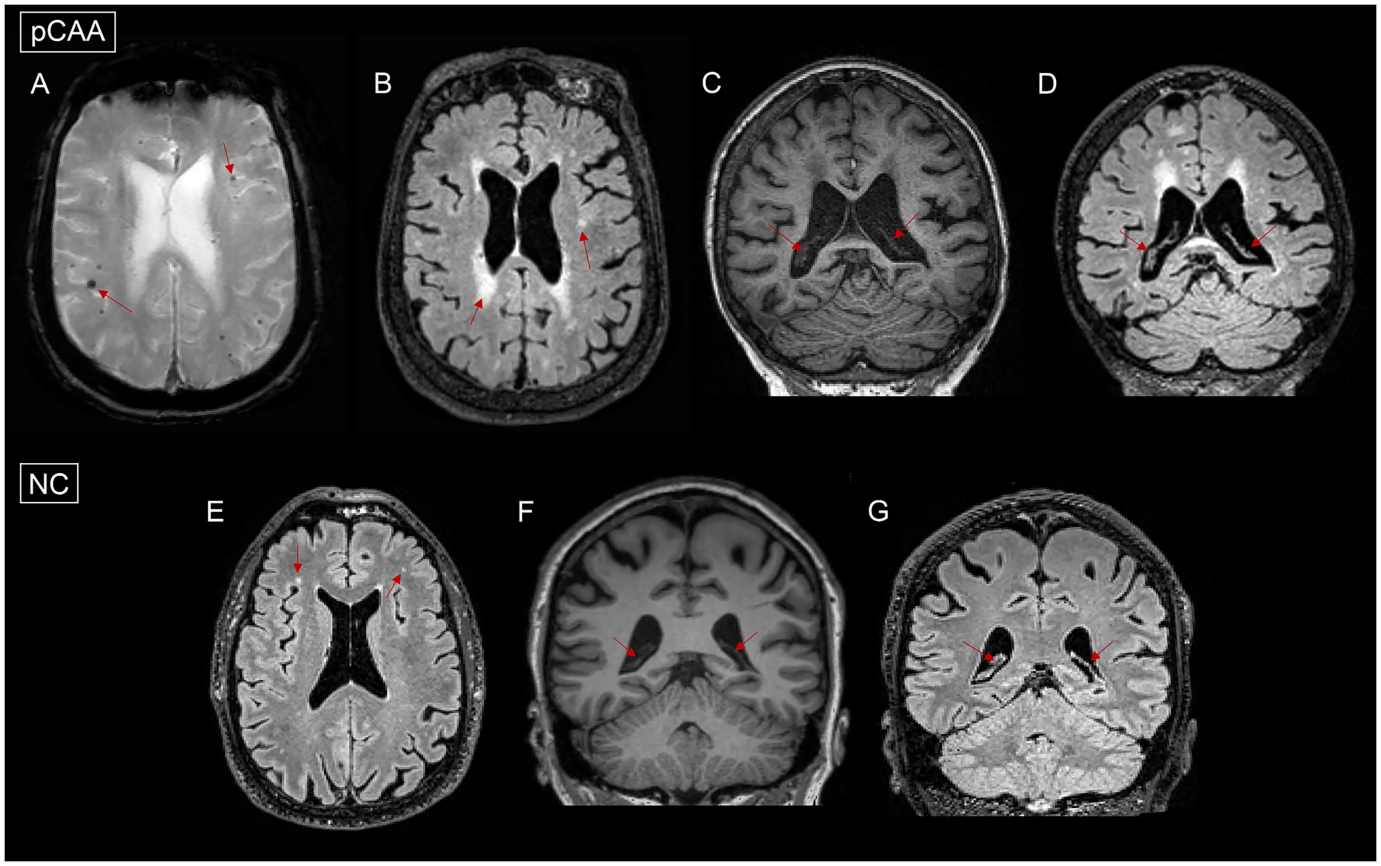
Images of microbleeds, white matter hyperintensities (WMH), and choroid plexus (ChP) in a normal control (NC) and a patient with probable cerebral amyloid angiopathy (pCAA). **(A)**: pCAA demonstrates microbleeds on T2*-weighted sequences. **(B)**: pCAA demonstrates white matter hyperintensities on T2 FLAIR sequences. **(C)**: pCAA demonstrates ChP on T1-weighted sequences. **(D)**: pCAA demonstrates ChP on T2 FLAIR sequences. **(E)**: NC demonstrates white matter hyperintensities on T2 FLAIR sequences. **(F)**: NC demonstrates ChP on T1-weighted sequences. **(G)**: NC demonstrates ChP on T2 FLAIR sequences.

Table 1 shows the demographics of the included subjects. Figure 2 briefly illustrates the imaging findings of normal controls and pCAA patients. There were no significant differences in age, sex, diabetes, hyperlipidemia, smoking, stroke, MI, AF, brain parenchymal-to-TIV ratio among the three groups (all *p* > 0.05).

**Table 1:** Demographic, clinical, and imaging data of the three groups.

Variable	NC (*N* = 26)	MCI-nonCAA (*N* = 26)	pCAA (*N* = 26)	*p* value
Demographic				
Age (years)	78.81 ± 6.14	78.19 ± 5.99	79.81 ± 7.40	0.669
Sex (female/total)	12/26	10/26	10/26	0.809
Vascular risk factors				
Hypertension, N (%)	9/26	8/26	17/26	0.022^b,c^
Diabetes, N (%)	3/26	3/26	3/26	1.000
Hyperlipidemia, N (%)	9/26	13/26	12/26	0.508
Smoking, N (%)	8/26	4/26	3/26	0.177
Stroke History, N (%)	1/26	0/26	0/26	0.363
MI, N (%)	2/26	5/26	4/26	0.477
AF, N (%)	1/26	1/26	3/26	0.425
Cognitive assessments				
MMSE	29.12 ± 1.28	26.92 ± 3.54	25.58 ± 2.90	<0.01^a,b^
MoCA	24.92 ± 2.71	21.62 ± 5.30	18.43 ± 5.81	<0.01^a,b,c^
CDR-SOB	0.17 ± 0.50	2.10 ± 2.09	3.14 ± 2.66	<0.01^a,b^
Brain damage indicators				
Microbleeds, N (IQR)	0[0–0]	0[0–0]	4[2–15]	<0.01^bc^
Cortical Superficial Siderosis, N (IQR)	0[0–0]	0[0–0]	0[0–0]	0.046^bc^
Deep WMH score				<0.01^b,c^
0	1/26	0/26	0/26	
1	18/26	22/26	4/26	
2	6/26	4/26	14/26	
3	1/26	0/26	8/26	
Periventricular WMH score				<0.01^b,c^
0	3/26	0/26	1/26	
1	17/26	17/26	3/26	
2	5/26	8/26	12/26	
3	1/26	1/26	10/26	
WMH volume, ml (IQR)	2.08 (0.92–5.49)	1.32 (0.61–4.57)	9.40 (4.72–24.8)	0.007^b,c^
WMH volume fraction, *10^−3^ (IQR)	1.50 (0.54–3.93)	0.85 (0.40–3.76)	6.33 (3.39–12.57)	0.034^b,c^
Parenchymal-to-TIV ratio	0.70 ± 0.39	0.70 ± 0.37	0.69 ± 0.05	0.799
AD pathological markers				
Aβ, Centiloid	17.5 ± 30.12	36.05 ± 44.37	67.30 ± 56.14	<0.01^b,c^
Aβ, positive, N (%)	10/24	10/22	17/20	0.007^b,c^
Tau, meta-ROI	1.12 ± 0.09	1.35 ± 0.28	1.52 ± 0.44	0.002^b^
Tau, positive, N (%)	10/24	7/19	12/20	0.001^b^
ChP volume (ml)	2.42 ± 0.76	2.34 ± 0.69	2.82 ± 1.00	0.095
ChP volume fraction, *10^−3^	1.59 ± 0.41	1.56 ± 0.39	1.77 ± 0.53	0.200
Mean DTI-ALPS	1.26 ± 0.16	1.19 ± 0.18	1.15 ± 0.44	<0.01^b^

WMH, White matter hyperintensity; NC, normal control; MCI, cognitive impairment; CAA, Cerebral amyloid angiopathy; AD, Alzheimer’s disease; TIV, Total intracranial volume; AF, auricular fibrillation; MI, Myocardial infarction.

^aNC vs. MCI-nonCAA; bNC vs. pCAA; cMCI-nonCAA vs. pCAA.^

Aβ data was available in 24 NC, 22 MCI-nonCAA, and 20 pCAA.

Tau data was available in 24 NC, 19 MCI-nonCAA, and 20 pCAA.

The NC, MCI-nonCAA, and pCAA groups exhibited significant differences in hypertension and cognitive assessments. For imaging markers, deep and periventricular WMH scores, WMH volume, and DTI-ALPS showed significant differences among the three groups. *Post hoc* analyses showed that patients with pCAA had significantly higher prevalence of hypertension, lower MMSE, lower MoCA, higher CDR-SOB, higher WMH burden, higher Aβ and tau deposition, and lower DTI-ALPS than NC. Compared to MCI-nonCAA, patients with pCAA showed significantly higher prevalence of hypertension, lower MoCA, higher WMH burden, and higher Aβ deposition. Although the pCAA group showed a trend of increased ChP volume, the difference was only marginally significant among the three groups.

In correlation analyses, we found that CSF circulation disorders were associated with downstream adverse events in the pCAA group. Specifically, we found a negative association (*r* = −0.478, *p* = 0.018) between DTI-ALPS and WMH volume fraction (Table 2). We did not observe that CSF circulation markers were associated with AD pathological markers. However, we found a negative association (*r* = −0.491, *p* = 0.024) between ChP volume fraction and MoCA (Table 2). After controlling for the VRFs, the findings remain consistent in additional correlation analyses (Table 3).

**Table 2:** Association between CSF circulation markers, brain degeneration and cognitive impairments in the pCAA group, controlling for age and sex.

	ChP volume fraction		DTI-ALPS	
	*r*	*p* value	*r*	*p* value
Brain volumetric markers				
WMH volume fraction	*0.246*	0.246	***−0.478***	**0.018**
Parenchymal-to-TIV ratio	*0.320*	0.128	*0.019*	0.930
AD pathological markers				
Aβ	*0.207*	0.410	*0.249*	0.319
Tau	*−0.023*	0.927	*0.171*	0.497
Cognitive function				
MMSE	*−0.256*	0.228	*−0.199*	0.352
MoCA	***−0.491***	**0.024**	*−0.095*	0.683
CDR-SOB	*0.018*	0.934	*0.168*	0.432

WMH, White matter hyperintensity; TIV, Total intracranial volume; AD, Alzheimer’s disease; MMSE, Mini-Mental State Examination; MoCA, Montreal Cognitive Assessment; CDR-SOB, clinical dementia rating scale sum of boxes. Bold: Significant at *p* < 0.05. Italics values are correlation coefficients.

**Table 3:** Association between CSF circulation markers, brain degeneration and cognitive impairments in the pCAA group, controlling for age, sex, and VRFs.

	ChP volume fraction		DTI-ALPS	
	*r*	*p* value	*r*	*p* value
Brain volumetric markers				
WMH volume fraction	*0.173*	0.429	***−0.606***	**0.002**
Parenchymal-to-TIV ratio	*0.248*	0.254	*−0.072*	0.743
AD pathological markers				
Aβ	*0.137*	0.601	*0.192*	0.460
Tau	*−0.018*	0.945	*0.181*	0.486
Cognitive function				
MMSE	*−0.239*	0.273	*−0.181*	0.408
MoCA	***−0.492***	**0.028**	*−0.081*	0.734
CDR-SOB	*−0.016*	0.944	*0.145*	0.510

WMH, White matter hyperintensity; TIV, Total intracranial volume; AD, Alzheimer’s disease; MMSE, Mini-Mental State Examination; MoCA, Montreal Cognitive Assessment; CDR-SOB, clinical dementia rating scale sum of boxes. Bold: Significant at *p* < 0.05. Italics values are correlation coefficients.

## Discussion

In this study, we examined two CSF circulation markers in NC, MCI-nonCAA, and pCAA groups. Our findings revealed that patients with pCAA had significantly lower DTI-ALPS values, higher WMH burden, more Aβ and tau deposition, and worse cognitive performance compared to NC. Within the pCAA group, a lower DTI-ALPS value was associated with a higher WMH burden, while a larger ChP volume correlated with a lower MoCA score. These results provide evidence of CSF circulation abnormalities in CAA, which may contribute to a better understanding of the disease’s etiology.

We screened over a thousand subjects in the ADNI3 database using the Boston 2.0 criteria for probable CAA and identified 26 cases. Unlike the high prevalence of CAA reported in pathological studies, imaging studies typically report a much lower prevalence (Jäkel et al., 2022). Additionally, variations in MRI scanners and protocols may affect the visualization of lesion features used to define CAA. The ADNI3 project acquired T2* images but did not include susceptibility-weighted imaging (SWI), further lowering the estimated prevalence (Buch et al., 2017). To disentangle the influence of cognitive status and CAA, we established two control groups. We applied a deep learning-based ChP segmentation method that utilizes two imaging modalities for segmentation. This method has a high segmentation accuracy compared to traditional tools.

We found significantly decreased DTI-ALPS and a trend of increased ChP volume for the pCAA group. As discussed before, the decreased DTI-ALPS indicated decreased fluid mobility in the perivenous space, while the increased ChP volume suggested pathological hypertrophy. This was in line with previous pathological and animal studies suggesting that CAA pathologies had a negative influence on CSF production and circulation. Nonetheless, when comparing to the MCI-nonCAA group, all results were non-significant, although the trends still existed. This is probably due to the small sample size and effect size.

The pCAA group had significantly higher WMH volume fraction and Aβ deposition compared to the MCI-nonCAA and NC groups. Regarding cognitive functions, the pCAA group showed lower cognitive scores in MoCA, but not MMSE and CDR, compared to the MCI-nonCAA group. These findings were consistent with the pathological theories of CAA, confirming that the Boston 2.0 diagnostic criteria of pCAA could correctly identify these patients.

In correlation analyses, we discovered that a lower DTI-ALPS was associated with a higher WMH burden in the pCAA group. Our previous studies demonstrated that venous disruption could cause stagnation of ISF and an increase in WMH volume (Zhang R. et al., 2021). The lower DTI-ALPS index, reflecting impaired perivenous fluid movement, may have similar effects. ISF stagnation can lead to the accumulation of metabolic waste products and pathological proteins, triggering inflammatory responses that further contribute to cellular damage, demyelination, and lesion formation (Abdul Hamid et al., 2024). Notably, increased ISF content itself may promote the development of WMH, as the FLAIR sequence is highly sensitive to changes in tissue water content. Additionally, we found that the ChP volume fraction was associated with MoCA score. It is possibly due to that ChP alterations disrupt brain homeostasis and accelerated brain degeneration (Godrich et al., 2022; Choi et al., 2023; Gokcal et al., 2021; Jo et al., 2021).

This research has several limitations. First, this is a cross-sectional study. The causal relationships between CSF circulation markers, parenchymal damage markers and cognitive decline over time still need to be explored in longitudinal studies. Second, we included pCAA without pathological confirmation. Some non-CAA participants might have been erroneously included. Furthermore, CAA can present with diverse clinical manifestations, including hemorrhagic stroke, transient focal neurological episodes, and cognitive impairment. Since our study exclusively enrolled patients with cognitive impairment, the generalizability of our findings to other CAA populations may be limited (Perini et al., 2023). Third, although we adjusted for age, sex, and vascular risk factors (VRFs), unmeasured confounding factors may still have influenced the findings. Fourth, T2*-GRE imaging was used to quantify microbleeds instead of the more sensitive SWI sequence, potentially contributing to a lower estimated prevalence. Fifth, the small sample size may have limited our ability to detect associations with smaller effect sizes.

## Conclusion

In conclusion, our study suggested that CSF circulation markers were associated with elevated WMH burden and cognitive impairments in probable CAA. Further validations in cohorts with large sample sizes are needed.

## Data Availability

The datasets presented in this study can be found in online repositories. The names of the repository/repositories and accession number(s) can be found at: https://adni.loni.usc.edu/.

## Footnotes

^1^ http://www.loni.ucla.edu/ADNI

^2^ http://adni.loni.usc.edu/

^3^ http://adni.loni.usc.edu/wp-content/uploads/2010/05/ADNI2_PET_Tech_Manual_0142011.pdf

^4^ https://surfer.nmr.mgh.harvard.edu/

^5^ http://www.fil.ion.ucl.ac.uk/spm

^6^ https://fsl.fmrib.ox.ac.uk/fsl

^7^ http://www.mrtrix.org

## ADNI consortia information

Data used in the preparation of this article were obtained from the Alzheimer’s disease Neuroimaging Initiative (ADNI) database (http://www.adni.loni.usc.edu). As such, the investigators within the ADNI contributed to the design and implementation of ADNI and provided data but did not participate in analysis or writing of this report. A complete listing of ADNI investigators can be found at https://adni.loni.usc.edu/wp-content/uploads/ADNI%20Acknowledgement%20List_Feb2025_clean.pdf. Full list of consortium members and affiliations for ADNI The full list of members and affiliations could be found in https://adni.loni.usc.edu/wp-content/themes/freshnews-dev-v2/documents/policy/ADNI%20Groups%20Acknowledgements%20Journal%20Format.pdf.
